# New records of helminths of *Sceloporus pyrocephalus* Cope (Squamata, Phrynosomatidae) from Guerrero and Michoacán, Mexico, with the description of a new species of *Thubunaea* Seurat, 1914 (Nematoda, Physalopteridae)

**DOI:** 10.3897/zookeys.716.13724

**Published:** 2017-11-24

**Authors:** Edgar Uriel Garduño-Montes de Oca, Jorge D. López-Caballero, Rosario Mata-López

**Affiliations:** 1 Departamento de Biología Evolutiva, Facultad de Ciencias, Universidad Nacional Autónoma de México. Avenida Universidad 3000, Ciudad Universitaria, C. P. 04510; Mexico City, Mexico; 2 Colección Nacional de Helmintos, Instituto de Biología, Universidad Nacional Autónoma de México. Avenida Universidad 3000, Ciudad Universitaria, C. P. 04510; Mexico City, Mexico; 3 Posgrado en Ciencias Biológicas, Universidad Nacional Autónoma de México, Apartado 70-153, C. P. 04510, Mexico City, Mexico

**Keywords:** Anoplocephalidae, Helminths, Heterakidae, Mesocestoididae, Pharyngodonidae, Physalopteridae, Reptilia, *Thubunaea*, Western Mexico.

## Abstract

A total of 61 specimens of the Red-headed Spiny Lizard *Sceloporus pyrocephalus* Cope (Phrynosomatidae) collected during the breeding season (June/July 2003, 2004 and 2005) from Western Mexico were examined for helminths. The morphological characterization of the helminths found was made through light microscopy and scanning electron microscopy. Nine taxa of helminths were identified, two cestodes: *Mesocestoides* sp. and *Oochoristica* sp., and seven nematodes: *Parapharyngodon ayotzinapaensis* Garduño-Montes de Oca, Mata-López & León-Règagnon, 2016, *Parapharyngodon tikuinii* Garduño-Montes de Oca, Mata-López & León-Règagnon, 2016, *Parapharyngodon* sp., Physalopterinae gen. sp., *Skrjabinoptera scelopori* Caballero-Rodríguez, 1971, *Strongyluris similis* Caballero, 1938 and a new species of *Thubunaea* Seurat, 1914. Larvae of *Mesocestoides* sp. and Physalopterinae gen. sp. were found in the body cavity and digestive tract, respectively. Excluding the species of *Parapharyngodon* Chatterji, 1933, *S. pyrocephalus* is recorded for the first time as a host of the remaining seven taxa of helminths. Additionally, *Thubunaea* *leonregagnonae* sp. n. is described and illustrated as a new nematode species, parasite of *S. pyrocephalus* from Mexico. This new species can be differentiated from the majority of its congeners by the absence of spicules, the particular pattern of caudal papillae in males and the small ratio of oesophagus length:male total body length (0.1–0.16).

## Introduction

Mexico is ranked as a country with the second highest diversity of reptiles, 864 species of which 493 are endemic to the country (Flores-Villela and García-Vázquez 2014). Lizards are the species-richest reptilian group with 417 species, and among them the Phrynosomatidae are the most diverse family (representing 15.9% of the total lizard diversity from Mexico). Despite this, our knowledge regarding the helminth fauna of this family is limited. Parasite records exist for only 15.3% of the total taxa within the Phrynosomatidae (Paredes-León et al. 2008).

*Sceloporus* Wiegmann (Phrynosomatidae) is a genus of New World lizards composed of 92 nominal species, of which 59 are endemic to Mexico (Uetz et al. 2016). Particularly, *Sceloporus pyrocephalus* Cope is an endemic lizard of Western Mexico associated with streams and rivers within tropical deciduous and semi-deciduous forest (Uetz et al. 2016), and distributed along the Pacific coast of the southwestern states of Jalisco, Colima, Michoacán and Guerrero as well as Central Mexico, and Southern Morelos. Despite some efforts to characterize the parasites of this species of lizard (Calisi et al. 2008), current knowledge of its helminth fauna is still far from being complete.

The purpose of the present study is to report on the helminth fauna of *S. pyrocephalus*, including the description of a new species of *Thubunaea* Seurat, 1914.

## Materials and methods

A total of 61 specimens of *S. pyrocephalus* were collected during the breeding season from June/July in 2003, 2004 and 2005 (permit FAUT-0056 issued to Virginia León-Règagnon by Secretaría del Medio Ambiente y Recursos Naturales, SEMARNAT). The specimens were captured by noosing or by hand in 12 localities from Michoacán state, and three localities from Guerrero state, Mexico (Table 1). These localities include tropical-wet and hot-semi-arid climates and elevations ranging from 18–1462 m a.s.l. Lizards were euthanized with an intraperitoneal overdose of pentobarbital sodium. The mouth, peritoneal cavity, and all internal organs were examined for helminths under a stereoscopic microscope. Helminths obtained were counted, fixed, and preserved following the procedure proposed by Lamothe-Argumedo (1997). Nematodes and cestodes were fixed in hot 4% and 10% formaldehyde solution, respectively, and stored in 70% ethanol. For morphological examination, nematodes were cleared in alcohol-glycerol, and mounted on temporary slides. Cestodes were stained with Mayer’s paracarmine and mounted on permanent slides using Canada balsam. Preserved specimens were observed under a light microscope. Original drawings were made with an Olympus BX53 microscope equipped with a drawing tube. For scanning electron microscopy (SEM), worms were dehydrated through a graded series of ethanol and then critical point dried with carbon dioxide, coated with a gold/palladium mixture using a Q150R Modular Coating System, and examined in a Hitachi S-2460N microscope and SU1015 SEM (Hitachi). Measurements of the new species are presented in micrometres, unless otherwise indicated; the range is followed by the mean in parentheses. Quantitative descriptors of parasite populations were calculated based on Bush et al. (1997). The helminths were deposited in the Colección Nacional de Helmintos (**CNHE**), Instituto de Biología (**UNAM**). Specimens of lizards were deposited in the Colección Herpetológica, Museo de Zoología, Facultad de Ciencias, UNAM (**MZFC-HE**) and the Collection of the Amphibian and Reptile Diversity Research Center at the University of Texas at Arlington (**ARDRC-UTA**).

**Table 1.** Sampling localities in Mexico for *Sceloporus pyrocephalus* examined in this study.

**Locality**	**GPS coordinates**	**Collection**	**Accession number**
Michoacán			
Maruata	18°16'29"N, 103°20'51"W	July 2004	58252 ^‡^, JAC-25491, JAC-25513
Caleta de Campos	18°04'48"N, 102°45'56"W	July 2004	58245^‡^, 58246^‡^, JAC-25529
Aquila	18°35'27.96"N, 103°34'0.12"W	July 2003	17445^†^-17447^†^
Nuevo Corongoros	19°06'14.5"N, 102°53'52"W	July 2005	18321^†^, 18322^†^, 18324^†^
Arteaga	18°38'48.48"N, 101°58'6.24"W	July 2005	18349^†^, JAC-26111, JAC-26112, JAC-26114, JAC-26115
La Huacana	18°40'24.2"N, 101°59'42.5"W	July 2005	18320^†^, 18343^†^-18348^†^
Los Laureles	18°58'14"N, 103°05'27"W	June 2004	58221^‡^, JAC-24752
San Isidro Tecuiluca	19°04'33"N, 102°53'37"W	June 2004	58232^‡^, 58235^‡^, JAC-24776, JAC-24778
Los Avillos	19°02'19"N, 102°58'28"W	June 2004	JAC-24755
Buenavista Tomatlán	19°10'35.8"N, 102°39'48.6"W	July 2005	18313^†^-18315^†^, 18317^†^, 18318^†^, 18332^†^, 18334^†^-18337^†^, 18340^†^, JAC-26102
Apatzingan	19°07'29"N, 102°24'05"W	July 2003	53473^‡^, 53474^‡^
Lombardia	19°10'35"N, 102°03'48"W	June 2004	58207^‡^, 58213^‡^, 58216^‡^, 58238^‡^, 58240^‡^, JAC-24718, JAC-24878
Guerrero			
Coyuquilla	17°17'48.12"N, 101°02'48.12"W	July 2004	JAC-25311
Tecpan de Galeana	17°10'37.20"N, 100°35'43.1"W	July 2005	JAC-25575, JAC-26136-JAC-26141
Coyuca de Catalán	17°59'41.2"N, 101°11'43.5"W	July 2004	JAC-25218

†MZFC-HE;

‡ARDRC-UTA; the specimens labeled as JAC are expected to be cataloged in the MZFC-HE.

## Results

A total of nine helminth taxa was found parasitizing *S. pyrocephalus*: two cestodes and seven nematodes (Table 2). Only *Mesocestoides* sp. and Physalopterinae gen. sp. were found as larval stages in the body cavity and digestive tract, respectively. The remaining taxa were found as adults located in the intestine. In the following section, we list the helminth species from *S. pyrocephalus* found during the present study, along with previous records of each parasite taxon in different *Sceloporus* spp. from Mexico, where applicable.

**Table 2.** Helminth taxa collected from *Sceloporus pyrocephalus* Cope (Phrynosomatidae) at various localities in Mexico. Values presented for 3 locality are number of hosts examined (n), followed by prevalence (P%) and mean abundance (MA – range or number of specimens recovered when number of hosts parasitized was 1).

Locality		Cestoda		Nematoda						
		*Mesocestoides* sp.^†, L^	*Oochoristica* sp.^‡, A^	*Parapharyngodon ayotzinapaensis* Garduño-Montes de Oca et al., 2016^§, A^	*Parapharyngodon tikuinii* Garduño-Montes de Oca et al., 2016^§, A^	*Parapharyngodon* sp.^§, A^	Physalopterinae gen. sp.^|, L^	*Skrjabinoptera scelopori* Caballero-Rodríguez, 1971^‡, A^	*Strongyluris similis* Caballero, 1938^§, A^	*Thubunaea leonregagnonae* sp. n.^|, A^
**Michoacán**										
Maruata	n							3	n=3	
	P%							100	33.33	
	MA							13.33 (7–24)	2.33 (7)	
Caleta de Campos	n							3	3	3
	P%							33.33	33.33	100
	MA							7.67 (23)	2.67 (8)	17.67 (9–23)
Aquila	n	3		3		3				
	P%	33.33		33.33		66.67				
	MA	42.33 (127)		5.66 (17)		1.33 (4)				
Nuevo Corongoros	n								3	
	P%								100	
	MA								6 (2–11)	
Arteaga	n			7	7	7				7
	P%			14.29	28.57	14.29				57.14
	MA			0.28 (2)	0.71 (2–3)	0.28 (2)				4.28 (3–18)
La Huacana	n				7			7		7
	P%				14.29			28.57		85.71
	MA				0.14 (1)			2 (1–13)		9.57 (1–17)
Los Laureles	n								4	
	P%								50	
	MA								4.75 (5–14)	
San Isidro Tecuiluca	n				5	5				
	P%				20	60				
	MA				0.2 (1)	0.6 (3)				
Los Avillos	n				1					
	P%				100					
	MA				– (1)					
Buenavista Tomatlán July 2005	n	22			22	22	22			
	P%	4.54			9.09	27.27	18.18			
	MA	0.13 (3)			0.23 (2–3)	1.14 (1–8)	11.32 (14–235)			
Apatzingan	n				2	2				
	P%				50	50				
	MA				0.5 (1)	0.5 (1)				
Lombardia	n		12	12	12	12	12			
	P%		8.3	16.67	16.67	16.67	8.33			
	MA		0.08 (1)	0.66 (2–4)	0.33 (1–3)	0.33 (4)	2.42 (29)			
**Guerrero**										
Coyuquilla	n			2	2					
	P%			50	50					
	MA			1.5 (3)	0.5 (1)					
Tecpan de Galeana	n			7		7				7
	P%			14.29		57.14				14.29
	MA			1.71 (12)		2.14 (3–4)				1 (7)
Coyuca de Catalán	n					1				
	P%					100				
	MA					– (4)				

Hosts parasitized/ hosts examined		2/61	1/61	6/61	11/61	20/61	5/61	6/61	7/61	14/61
Specimens recovered		130	1	42	19	58	278	77	52	157
Intensity range		3–27	–	2–17	1–5	1–25	29–249	14–40	7–19	7–67

Site in host: † Body cavity; ‡ Small intestine; § Large intestine; | Stomach. L = larval stage; A = adult stage.

### Phylum Platyhelminthes Gegenbaur, 1859

#### Class Cestoda Rudolphi, 1808

##### Family Mesocestoididae Fuhrmann, 1907

###### Genus *Mesocestoides* Vaillant, 1863

####### Mesocestoides

Taxon classificationAnimaliaCyclophyllideaMesocestoididae

sp.

######## Specimens deposited.

CNHE 9464, 9465.

######## Other hosts.

*Sceloporus jarrovi* Cope in Chihuahua, Morelos and San Luis Potosí (Goldberg et al. 1996); *S. grammicus* Wiegmann in Mexico City (Goldberg et al. 2003); *S. torquatus* Wiegmann in Querétaro (Goldberg et al. 2003).

######## Remarks.

Four species of *Mesocestoides* are distributed in Mexico in carnivorous mammals: *M. bassarisci* MacCallum, 1921 in *Bassariscus astutus* Lichtenstein (Procyonidae) and *M. lineatus* (Goeze, 1782) in *Mephitis macroura* Lichtenstein (Mephitidae), both from Guerrero; *M. variabilis* Mueller, 1928 and *M. vogae* Etges, 1991 in *Canis lupus familiaris* Linnaeus (Canidae) from Mexico City (Paredes-León et al. 2008). Unfortunately, the specimens found in *S. pyrocephalus* could not be identified to species level because they were at the tetratiridium larval stage. Molecular analyses are needed to determinate the species identity of the larvae found in reptilian hosts, which serve as intermediate hosts (Santoro et al. 2012).

##### Family Anoplocephalidae Cholodkovsky, 1902

###### Genus *Oochoristica* Lühe, 1898

####### Oochoristica

Taxon classificationAnimaliaCyclophyllideaAnoplocephalidae

sp.

######## Specimens deposited.

CNHE 9469.

######## Remarks.

Eight species of *Oochoristica* have been recorded in Mexico: *O. acapulcoensis* Brooks, Pérez-Ponce de León & García-Prieto, 1999; *O. leonregagnonae* Arizmendi-Espinosa, García-Prieto & Guillén-Hernández, 2005; *O. osheroffi* Meggitt, 1934 and *O. whitentoni* Stellman, 1939, all parasites of *Ctenosaura pectinata* Weigmann (Iguanidae) from Acapulco, Guerrero (Brooks et al. 1999), Mixtequilla, Oaxaca (Arizmendi-Espinoza et al. 2005), Alpuyeca, Morelos (Flores-Barroeta et al. 1960), and Iguala, Guerrero (Flores-Barroeta 1955). *Oochoristica whitfieldi* Guillén-Hernández, García-Prieto & Arizmendi-Espinosa, 2007 was found parasitizing *C. oaxacana* Köhler & Hasbun (Iguanidae) (Guillén-Hernández et al. 2007); *O. parvula* Stunkard, 1938 was found in *Coleonyx elegans* Gray (Eublepharidae) from Oxkutzcab, Yucatán (Stunkard 1938); *O. phrynosomatis* Harwood, 1932 in *Phrynosoma braconnieri* Dugès, and *P. taurus* Duméril & Bocourt (Phrynosomatidae) from Cacaloapan and Caltepec, both in Puebla (Goldberg and Bursey 1991). *Oochoristica scelopori* Voge & Fox, 1950 was found infecting species of the family Phrynosomatidae: *S. jarrovi* (Goldberg et al. 1996); *S. parvus* Smith, *S. grammicus*, *S. megalepidurus* Smith, *S. variabilis* Wiegmann, *S. mucronatus* Cope, and *P. ditmarsi* Stejneger (Goldberg et al. 2003). Only one adult specimen of *Oochoristica* was found in the present study; however, it was not identified to specific level due to the absence of gravid proglottids.

### Phylum Nematoda Cobb, 1932

#### Class Chromadorea Inglis, 1983

##### Family Pharyngodonidae Travassos, 1920

###### Genus *Parapharyngodon* Chatterji, 1933

####### Parapharyngodon ayotzinapaensis

Taxon classificationAnimaliaOxyuridaPharyngodonidae

Garduño-Montes de Oca, Mata-López & León-Règagnon, 2016

######## Specimens deposited.

CNHE 9432–9438.

######## Remarks.

Eleven species of *Parapharyngodon* have been recorded in Mexico (Garduño-Montes de Oca et al. 2016), eight of them endemic, representing 10% of the world diversity of this genus. The high species richness of *Parapharyngodon* is probably related to the geographical and environmental heterogeneity of this region, and was recently revealed by parasitological surveys of host species not considered in previous studies (Jiménez et al. 2008, Bursey and Goldberg 2015, Velarde-Aguilar et al. 2015, Garduño-Montes de Oca et al. 2016).

####### Parapharyngodon tikuinii

Taxon classificationAnimaliaOxyuridaPharyngodonidae

Garduño-Montes de Oca, Mata-López & León-Règagnon, 2016

######## Specimens deposited.

CNHE 9439–9447.

######## Remarks.

See *P. ayotzinapaensis* remarks.

####### Parapharyngodon

Taxon classificationAnimaliaOxyuridaPharyngodonidae

sp.

######## Specimens deposited.

CNHE 9448–9454, 9470, 9471.

######## Remarks.

Female specimens of *Parapharyngodon* sp. were recovered from hosts in the same localities as *P. ayotzinapaensis* and *P. tikuinii*. The almost identical morphology of females in both species did not allow us to discriminate between them on species level (Garduño-Montes de Oca et al. 2016).

##### Family Heterakidae Railliet & Henry, 1912

###### Genus *Strongyluris* Müller, 1894

####### Strongyluris similis

Taxon classificationAnimaliaAscarididaHeterakidae

Caballero, 1938

######## Specimens deposited.

CNHE 9455–9459.

######## Other hosts.

*S. torquatus* in Mexico City (Cid del Prado 1971); *S. jarrovi* in Durango, Guanajuato, Hidalgo, Morelos, San Luis Potosí, Sinaloa, Sonora, Tamaulipas and Veracruz (Goldberg et al. 1996); *S. grammicus* and *S. mucronatus* in localities not further specified (Goldberg et al. 2003); *S. formosus* Wiegmann in Oaxaca (Goldberg et al. 2003).

######## Remarks.

Specimens recovered during the present study share certain morphological characters with *S. panamaensis* Bursey, Goldberg & Telford, 2003 and *S. similis* such as spicule length and number of caudal papillae. Our specimens were identified as *S. similis* since they possess two pairs of lateral subterminal papillae at the base of the caudal appendage (Caballero 1938), which is a diagnostic feature of this species, contrary to the three pairs of papillae observed at the base of the caudal appendage in *S. panamaensis* (Bursey et al. 2003).

##### Family Physalopteridae (Railliet, 1893) Leiper, 1908

###### Subfamily Physalopterinae Railliet, 1893

####### Physalopterinae

Taxon classificationAnimaliaSpiruridaPhysalopteridae

gen. sp.

######## Specimens deposited.

CNHE 9466–9468.

######## Other hosts.

*Abbreviata terrapenis* Hill, 1941 in *S. jarrovi* from Tamaulipas (Goldberg et al. 1996). *Physaloptera retusa* Rudolphi, 1819 in *S. jarrovi* from Aguascalientes, Chihuahua, Coahuila, Durango, Guanajuato, Morelos, Nuevo León, Querétaro, San Luis Potosí, Sinaloa, Sonora and Tamaulipas (Goldberg et al. 1996); in *S. acanthinus* Bocourt from Motozintla, Chiapas (Caballero 1951); in *S. jarrovi* (Goldberg et al. 1996) and *S. parvus* (Goldberg et al. 2003) from Hidalgo; in *S. formosus* from Oaxaca (Goldberg et al. 2003); in *S. mucronatus* from Puebla (Goldberg et al. 2003); in *S. torquatus* from Zacatecas (Goldberg et al. 2003). *Skrjabinoptera phrynosoma* (Ortlepp, 1922) Schulz, 1927 in *S. jarrovi* from Guanajuato (Goldberg et al. 1996); in *S. spinosus* Wiegmann from Actopan, Hidalgo (Caballero 1937); in *S. jarrovi* from Querétaro (Goldberg et al. 1996), and finally, in *S. grammicus* and *S. variabilis* from localities not further specified (Goldberg et al. 2003).

######## Remarks.

Representatives of the subfamily Physalopterinae use ants and beetles as intermediate hosts, which are part of the diet of *S. pyrocephalus*. By eating that sort of prey, this group of lizards becomes a potential intermediate or paratenic host of these nematodes (Petri 1950, Schell 1952, Lee 1957, Kabilov 1980).

###### Genus *Skrjabinoptera* Schulz, 1927

####### Skrjabinoptera scelopori

Taxon classificationAnimaliaSpiruridaPhysalopteridae

Caballero-Rodríguez, 1971

######## Specimens deposited.

CNHE 9460–9463.

######## Other hosts.

*S. grammicus* in San Andrés Totoltepec and San Ángel, Mexico City (CNHE); *S. torquatus* in Mexico City (Caballero-Rodríguez 1971). *Skrjabinoptera* sp. in *S. torquatus* from San Ángel, Mexico City (Cid del Prado 1971).

######## Remarks.

*Skrjabinoptera* is a genus of nematodes poorly represented around the world with only 10 species described as parasites, mainly of lizards, and only one species recorded from a snake (Rudolphi 1819). In Mexico, only *S. scelopori* has been recorded in three species of lizards: *S. torquatus*, *S. grammicus*, and *Phyllodactylus lanei* Smith (Gekkonidae) (Goldberg and Bursey 2000, Paredes-León et al. 2008). *Sceloporus pyrocephalus* is the fourth host species recorded for this helminth in the country.

##### Subfamily Thubunaeinae Sobolev, 1949

###### Genus *Thubunaea* Seurat, 1914

####### Thubunaea leonregagnonae sp. n.

Taxon classificationAnimaliaSpiruridaPhysalopteridae

http://zoobank.org/E2747F2B-1083-45B4-876C-D83E13A9F3AA

Figs 1A–H ; 2A–F

######## Type host.

*Sceloporus pyrocephalus* Cope (Squamata: Phrynosomatidae).

######## Symbiotype.

MZFC-HE 18345

######## Type locality.

Los Pocitos, La Huacana, Michoacán, Mexico (18°40'24.2"N, 101°59'42.5"W). Collected on July 7, 2005.

######## Site in host.

Stomach.

######## Prevalence and intensity of infection.

23% (14 of 61 hosts examined), with a mean intensity of 11 (7–67).

######## Type specimens.

Holotype: CNHE 9426 (1 male); allotype: CNHE 9427 (1 female); paratypes CNHE 9428, 9429, 9430 and 9431 (9 females and 7 males).

######## Etymology.

This species is named in honour of Virginia León-Règagnon (Instituto de Biología, UNAM), who was the mentor of the authors of this paper, and for her valuable contribution to our knowledge of helminth parasites in Mexico.

######## General description.

**Figure 1.**
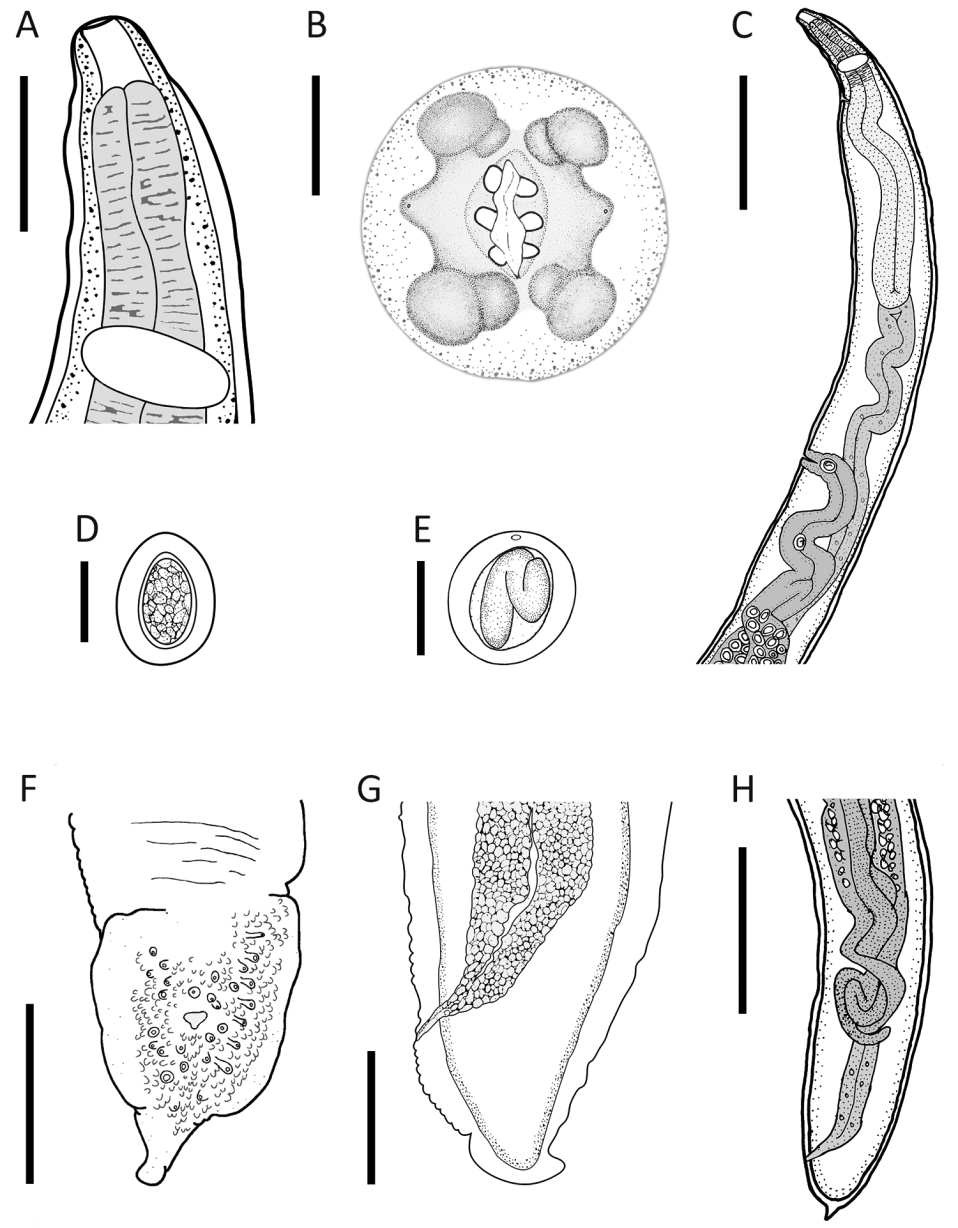
*Thubunaea leonregagnonae* sp. n. **A** Anterior end, female, lateral view **B** Apical view, female **C** Anterior end, female, lateral view showing excretory pore and vulva **D** Embryonated egg, lateral view **E** Larvated egg, lateral view **F** Caudal end, male, ventral view **G** Caudal end, male, lateral view, caudal papillae and ornamentation not shown **H** Caudal end, female, lateral view. Scale bars: **A** 90 µm; **B** 20 µm; **C** 370 µm; **D** 25 µm; **E** 30µm; **F** 250 µm; **G** 50 µm; **H** 370 µm.

**Figure 2.**
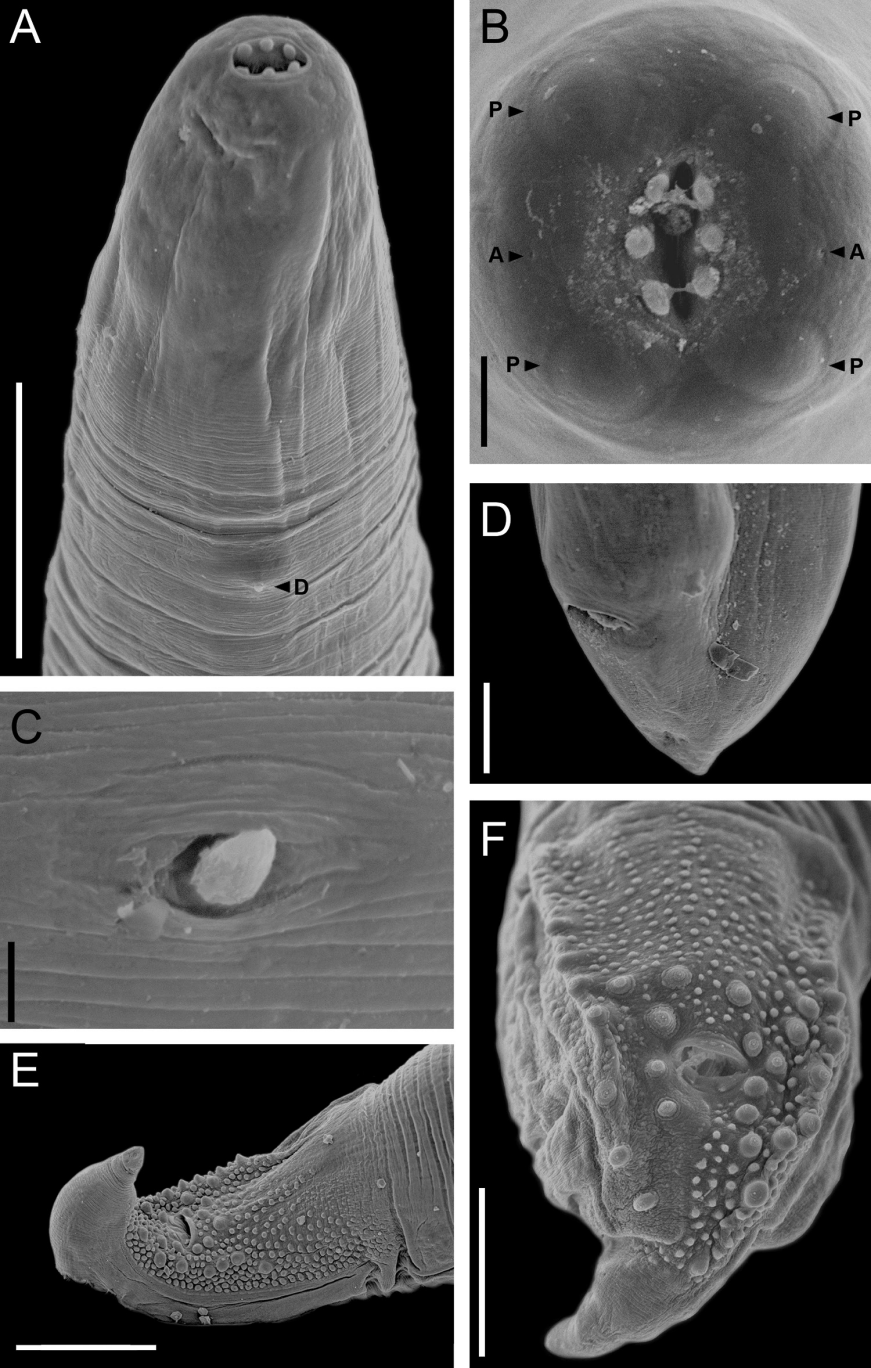
*Thubunaea leonregagnonae* sp. n. Scanning electron micrographs. **A** Anterior end, male, lateral view showing deirid (**D**) **B** Apical view, female, showing sub-median papillae (**P**) and amphids (**A**) **C** Deirid, lateral view **D** Posterior end, female, ventrolateral view **E** Posterior end, male, ventral view **F** Caudal end, male, ventral view. Scale bars: **A** 50 µm; **B** 10 µm; **C** 2.5 µm; **D** 50 µm; **E** 100 µm; **F** 50 µm.

Medium-sized nematodes, filiform body, cuticle with fine transverse striations along entire body. Males smaller than females. Round cephalic plate in both sexes (Figs 1B, 2B). Deirids symmetrical, simple with rounded tip (Fig. 2A, C), located immediately posterior to nerve ring. Mouth with two round and simple lateral lips, each with three small teeth on its internal surface; each lip bears a lateral amphid, and a pair of sub-median papillae (Figs 1B, 2B). Pharynx short, cylindrical, opening into oesophagus. Oesophagus divided into anterior muscular portion and posterior glandular portion. Excretory pore in anterior region of body, posterior to nerve ring and located at level of division of muscular and glandular oesophagus. Posterior end conical and rounded in both sexes (Figs 1F, H, 2D, F).

**Description of male** (based on eight specimens; the number of measurements, where different from eight, is given in parentheses): Total body length (MTBL) 4.85–8.03 mm (6.19), width at mid-body 200–300 (243). Deirids 150–185 (166; n = 7) from anterior end. Nerve ring and excretory pore 100–168 (125) and 125–295 (179, n = 6) from anterior end, respectively. Pharynx length 23–45 (33). Oesophagus total length 613–1038 (800, n = 7), muscular portion length 108–170 (128, n = 7), glandular portion length 488–913 (671, n = 7). Ratio oesophagus total length: MTBL 0.1–0.16 (0.13, n = 7). Testis elongated, distributed in zigzag from anterior intestinal portion to posterior region, near cloaca. Caudal alae well developed, bearing ventrally numerous papilliform plates. Cloaca surrounded by numerous papillae (24–28), 11–14 sessile papillae and 10–16 pedunculate papillae distributed asymmetrically in the following arrangement: ventrolateral: 4–8 pedunculate on right, 6–9 pedunculate on left side; ventral, sessile: 5–8 on right and 4–8 on left side (Figs 1F, 2E, F). Number and disposition of papillae variable with respect to cloaca, thus, precloacal, paracloacal, and postcloacal positions not established. Spicules and gubernaculum absent. Tail 50–67 (59, n = 6) long.

**Description of female** (based on ten gravid specimens; the number of measurements, where different from ten, is given in parentheses): Total body length (FTBL) 8.72–21.46 mm (14.61), width at mid-body 270–440 (349). Deirids 125–250 (173, n = 8) from anterior end. Nerve ring and excretory pore 100–188 (147, n = 8) and 193–343 (256, n = 6) from anterior end, respectively. Pharynx length 30–58 (41, n = 9). Oesophagus total length 853–1500 (1227, n = 7); muscular portion length 125–325 (220, n = 8), glandular portion length 703–1220 (996, n = 7); ratio oesophagus total length:FTBL 0.1–0.16 (0.14, n = 7). Didelphic, opisthodelphic, ovaries distributed in posterior region of body, uteri extended parallel along almost entire body. Vagina muscular, directed posteriorly, located in anterior region of body close to anterior end of intestine, vulva at 1130–2570 (1770) from anterior end (Fig. 1C). Ratio distance vulva to anterior end of body: FTBL 0.11–0.13 (0.12). Tail 25–30 (27, n = 8) long. Embryonated eggs occupying almost entire uterus, thick shelled with smooth surface, 39–44 (41, n = 15) long by 29–33 (31, n = 15) wide (Fig. 1D); larvated eggs located near vulva, 45–50 (48, n = 15) long by 38–40 (39, n = 15) wide (Fig. 1E).

######## Remarks.

The family Physalopteridae is composed of three subfamilies: Thubunaeinae Sobolev, 1949, Proleptinae Schulz, 1927 and Physalopterinae Railliet, 1893 (Chabaud 1975). Thubunaeinae comprises two genera *Thubunaea* and *Physalopteroides* Wu & Liu, 1940, both of which are parasites of reptiles and are characterized by the absence of a cephalic ring, the presence of numerous caudal papillae, and an ornamented cuticle forming papillary plates distributed on the surface of the cauda in males (Chabaud 1975). These two genera differ from each other mainly by the symmetry of their cephalic structures; in *Thubunaea* these structures are symmetrical, while they are asymmetrical in *Physalopteroides* (Chabaud 1975). Some authors, for example Moravec et al. (1997), considered that the morphological features of *Thubunaea* and *Physalopteroides* have rarely been analysed using techniques such as SEM, and that these observations could provide detailed information to assess the validity of these two genera. However, to the best of our knowledge, SEM studies are still scarce in both genera, being available for only three species of *Thubunaea* (Moravec et al. 1997, Pazoki and Rahimian 2014, Ramallo et al. 2016) and two species for *Physalopteroides* (Elwasila 1990, Goswami et al. 2016). The specimens described in the present study show a symmetrical cephalic structure, as in *Thubunaea*.

Currently, 20 species of *Thubunaea* are considered as valid: one in the Afrotropical region, *T. fitzsimonsi* Ortlepp, 1931; five in the Nearctic region, *T. cnemidophorus* Babero & Matthias, 1967, *T. ctenosauri* Moravec, Salgado-Maldonado & Mayen-Peña, 1997, *T. iguanae* Telford, 1965, *T. intestinalis* Bursey & Goldberg, 1991 and *T. leiolopismae* Harwood, 1932; two in the Neotropics, *T. parkeri* Baylis, 1926, and *T. eleodori* Ramallo, Goldberg, Bursey, Castillo & Acosta, 2016; six in the Oriental region, *T. aurangabadensis* Deshmukh, 1969, *T. brooki* Deshmukh, 1969, *T. hemidactylae* Oshmarin & Demshin, 1972, *T. mirzai* Narayan, 1941, *T. singhi* Deshmukh, 1969, and *T. syedi* Deshmukh, 1969; two in the Palearctic region, *T. schukurovi* Annaev, 1973 and *T. smogorzhewskii* Sharpilo, 1966; and four species in the Saharo-Arabian region, *T. baylisi* Akhtar, 1939, *T. dessetae* Barus & Tenora, 1976, *T. mobedii* Pazoki & Rahimian, 2014 and *T. pudica* Chabaud & Golvan, 1957 (Ramallo et al. 2016).

Six of these species lack spicules, as is the case in *T. leonregagnonae* sp. n.: *T. cnemidophorus*, *T. eleodori*, *T. fitzsimonsi*, *T. mobedii*, *T. parkeri* and *T. schukurovi* (Table 3). Males of four of these species (*T. eleodori*, *T. fitzsimonsi*, *T. parkeri* and *T. mobedii*) are larger in body length than *T. leonregagnonae* sp. n. (10.25–11.16, 8.5–9, 10.5, 11.4–15.4 vs 4.85–8.03 mm, respectively). In the number of papillae, pedunculate or sessile, the new species also differs from most of these six species. *Thubunaea leonregagnonae* sp. n. has 10–16 pedunculate papillae and 11–14 sessile papillae, *T. mobedii* does not have pedunculate papillae; in contrast, *T. fitzsimonsi* and *T. parkeri* lack sessile papillae, and in this latter species the number of caudal papillae is smaller (16–20) than in the present specimens (24–28) (Table 3). *Thubunaea leonregagnonae* sp. n. is most similar to *T. cnemidophorus*, *T. eleodori* and *T. schukurovi*, however, it can be distinguished from the last two species in the number of sessile papillae: *T. eleodori* has ten and *T. schukurovi* has 16, instead of 11–14 in *T. leonregagnonae* sp. n. Males of *T. leonregagnonae* sp. n. and *T. cnemidophorus* can be differentiated mainly by having a different oesophagus length/MTBL ratio (0.1–0.16 vs 0.26) and by the former having a smaller body width (200–300 vs 350–390).

**Table 3.** Morphological characters of *Thubunaea* spp. lacking spicules in males. Measurements in micrometres, unless otherwise indicated.

Species	*T. cnemidophorus* Babero & Matthias, 1967	*T. eleodori* Ramallo, Goldberg, Bursey, Castillo & Acosta, 2016	*T. fitzsimonsi* Ortlepp, 1931	*T. mobedii* Pazoki & Rahimian, 2014	*T. parkeri* Baylis, 1926	*T. schukurovi* Annaev, 1973	*Thubunaea leonregagnonae* sp. n.
Source	Babero and Matthias (1967)	Ramallo et al. (2016)	Ortlepp (1931)	Pazoki and Rahimian (2014)	Baylis (1926)	Annaev (1973)	Present study
Type host	*Aspidoscelis tigris* (Baird & Girard) (Teiidae)	*Liolaemus eleodori* Cei, Etheridge & Videla (Liolaemidae)	*Meroles squamulosus* (Peters) (Lacertidae)	*Laudakia nupta nupta* (De Filippi) (Agamidae)	*Microlophus occipitalis* (Peters) (Tropiduridae)	*Ablepharus deserti* Strauch (Scincidae)	*Sceloporus pyrocephalus* Cope (Phrynosomatidae)
Country	USA	Argentina	South Africa	Iran	Peru	Turkmenistan	Mexico
Biogeographical realm	Nearctic	Neotropical	Afrotropical	Saharo-Arabian	Neotropical	Palearctic	Nearctic
MTBL (mm)	5.7–6.6	10.25–11.16	8.5–9.0	11.4–15.4	10.5	7.8	4.85–8.03
MTBW	350–390	360	290–330	190–330	300–340	300	200–300
PP	14–16	12	29–31	0	16–20	12	10–16
SP	12	10	0	23–37	0	16	11–14
OE/MTBL	0.26^†^	0.12	0.11	0.12	0.1	0.15	0.1–0.16
Eggs	38–45 × 23–30			44–45 × 36–39	58–63 × 48–53		45–50 × 38–40
	(38 x 23)	(48 x 41)	(45 x 38)	(48 x 38)	(60 x 50)	(43 x 33)	

MTBL: male total body length (mm); MTBW: male total body width; PP: number of pedunculate papillae; SP: number of sessile papillae; OE: oesophagus length. † data from Bursey and Goldberg (1991).

## Discussion

Many authors have argued that the inventory of the parasite fauna of host species is of critical importance in biodiversity management and conservation efforts (Brooks and Hoberg 2000, Brooks and McLennan 2002, Funks and Richardson 2002). Parasite studies help to reveal other biological aspects of the hosts such as their natural history and ecology. The close link between parasites and their hosts causes these studies to have a cascade effect on our knowledge of the biology of the interaction between host and parasite, as well as with the environment in which this association developed. For this reason, helminthological studies on hosts distributed in areas with high potential for endemism, such as in the present study, are highly relevant for understanding the ecology and evolution of parasite-host interactions.

Presently, for *S. pyrocephalus*, a single study was conducted, evaluating parasite load in conjunction with hormone concentration (Calisi et al. 2008). In this study, only nematodes and cestode larvae were found, without, however, identifying these to species level.

In the present study, nine helminth taxa were found parasitizing *S. pyrocephalus*: two tapeworms of the order Cyclophyllidea (*Mesocestoides* sp. and *Oochoristica* sp.), one nematode of the order Ascaridida (*S. similis*), three of the order Rhabditida (*P. ayotzinapaensis*. *P. tikuinii*, and *Parapharyngodon* sp.), and three of the order Spirurida (Physalopterinae gen. sp., *S. scelopori* and *T. leonregagnonae* sp. n.). Most of these taxa coincide with previous reports on the helminth fauna from lizards in Mexico (Caballero 1938, Telford 1965, Goldberg et al. 1996, Moravec et al. 1997, Goldberg et al. 2003, Paredes-León et al. 2008). *Parapharyngodon ayotzinapaensis*, *P. tikuinii* and *T. leonregagnonae* sp. n. have been recorded as specific nematode species for this lizard. The current results increase from 35 to 40 the number of helminths recorded as parasites of *Sceloporus* spp. and from 17 to 18 the number of species of this lizard genus for which parasite records are available (Paredes-León et al. 2008).

*Oochoristica* sp., *P. ayotzinapaensis*, *P. tikuinii*, *Parapharyngodon* sp., *S. scelopori*, *S. similis*, and *T. leonregagnonae* sp. n. use *S. pyrocephalus* as a definitive host, and *Mesocestoides* sp. and Physalopterinae gen. sp. were recorded as larval stages. *Mesocestoides* sp. is a common metacestode found in the body cavity and mesentery of amphibians and reptiles, which serve as paratenic hosts, with carnivorous mammals being the definitive hosts (Santoro et al. 2012). Specimens of Physalopterinae gen. sp. were found in the stomach and large intestine; species belonging to this subfamily are adult parasites of amphibians, reptiles and mammals, so their presence in *S. pyrocephalus* was probably a result of a recent recruitment.

It has been suggested that reptiles that use a passive feeding strategy (i.e. sit-and-wait) have a less diverse and less complex helminth fauna than those with an active searching behaviour (e.g. widely foraging) (Roca 1999), and that these foraging strategies are directly related to the diet of the lizards, with a passive strategy used by predominantly herbivorous lizards and an active search used by carnivorous ones. Nematodes with a direct life cycle, such as pinworms (Oxyurida: Pharyngodonidae), best reveal the differences between herbivore and carnivore hosts. Within the Pharyngodonidae, a family of Oxyuroidea that are characteristic parasites of amphibians and reptiles, are two evolutionary lineages that show a diversification that mirrors that of their hosts' diets. (Roca 1999). Some genera of Pharyngodonidae infect only carnivorous saurian reptiles: *Parapharyngodon*, *Spauligodon* Skrjabin, Schikhobalova & Lagodovskaja, 1960, *Skrjabinodon*, *Pharyngodon* and *Parathelandros* Baylis, 1930; while the second linage is composed of pharyngodonid parasites of herbivorous iguanids and testudines: *Tachygonetria* Wedl, 1862, *Mehdiella* Seurat, 1918, *Alaeuris* Seurat, 1918, *Thaparia* Ortlepp, 1933, *Ortleppnema* Petter, 1966, *Ozolaimus* Dujardin, 1845, *Travassozolaimus* Vigueras, 1938 and *Mamillomacracis* Dosse, 1939. Given the particular species of Oxyuroidea found in *S. pyrocephalus*, along with the presence of *S. similis*, a typical nematode found in carnivorous reptiles (Núñez 2005), it can be inferred that this lizard is mainly carnivorous. The remaining taxa of helminths found in the present study, including the nematodes *T. leonregagnonae* sp. n. and *S. scelopori*, have an indirect life cycle, in which beetles and ants are probably acting as intermediate hosts. Studies in other species of *Sceloporus* have determined that termites are an important food source for *S. gadoviae* Boulenger, *S. horridus* Wiegmann, and *S. jalapae* Günther (Serrano-Cardozo et al. 2008); hence, termites could likely be the intermediate hosts of the heteroxenous parasites found in *S. pyrocephalus*.

Since five of the nine parasite taxa recorded for *S. pyrocephalus* have an indirect life cycle, with arthropods as intermediate hosts, diet might be the predominant factor structuring the helminth fauna in this lizard. This opposes the idea proposed by Aho (1990), who found that most of the parasite species that inhabit the intestine of amphibians and reptiles, are nematodes with a direct life cycle. Studies assessing the helminth fauna associated with Mexican reptiles are of great value to have a better understanding of the factors that influence the ecological dynamics within helminth communities and to establish comparisons between Nearctic and Neotropical populations of hosts.

## Supplementary Material

XML Treatment for Mesocestoides

XML Treatment for Oochoristica

XML Treatment for Parapharyngodon ayotzinapaensis

XML Treatment for Parapharyngodon tikuinii

XML Treatment for Parapharyngodon

XML Treatment for Strongyluris similis

XML Treatment for Physalopterinae

XML Treatment for Skrjabinoptera scelopori

XML Treatment for Thubunaea leonregagnonae
